# MicroRNA Expression Profiles of Epicardial Adipose Tissue-Derived Exosomes in Patients with Coronary Atherosclerosis

**DOI:** 10.31083/j.rcm2306206

**Published:** 2022-05-31

**Authors:** Jinxing Liu, Ang Gao, Yan Liu, Yan Sun, Dai Zhang, Xuze Lin, Chengping Hu, Yong Zhu, Yu Du, Hongya Han, Yang Li, Shijun Xu, Taoshuai Liu, Chenhan Zhang, Junming Zhu, Ran Dong, Yujie Zhou, Yingxin Zhao

**Affiliations:** ^1^Department of Cardiology, Beijing Anzhen Hospital, Capital Medical University, Beijing Institute of Heart Lung and Blood Vessel Diseases, Beijing Key Laboratory of Precision Medicine of Coronary Atherosclerotic Disease, Clinical center for coronary heart disease, Capital Medical University, 100029 Beijing, China; ^2^Department of Cardiology, State Key Laboratory of Cardiovascular Disease, Fuwai Hospital, National Center for Cardiovascular Diseases, Chinese Academy of Medical Sciences and Peking Union Medical College, 100037 Beijing, China; ^3^Department of Cardiac Surgery, Beijing Anzhen Hospital, Capital Medical University, 100029 Beijing, China

**Keywords:** epicardial adipose tissue, exosomes, microRNAs, atherosclerosis, coronary artery disease

## Abstract

**Background and Aims:**

Epicardial adipose tissue, exosomes, and miRNAs have important activities in atherosclerosis. The purpose of this study was to establish miRNA expression profiles of epicardial adipose tissue-derived exosomes in patients with coronary atherosclerosis.

**Methods:**

Biopsies of epicardial adipose tissue were obtained from patients with and without coronary artery disease (CAD, n = 12 and NCAD, n = 12) during elective open-heart surgeries. Tissue was incubated with DMEM-F12 for 24 hours. Exosomes were isolated, then nanoparticle tracking analysis, transmission electron microscopy, and immunoblotting were performed to confirm the existence of exosomes. Total RNA in exosomes was subjected to high-throughput sequencing to identify differentially expressed miRNAs. MicroRNA target gene prediction was performed, and target genes were analyzed by Gene Ontology (GO), the Kyoto Encyclopedia of Genes and Genomes (KEGG), and mirPath to identify function. Reverse transcription quantitative PCR was performed to confirm the differentially expressed miRNAs.

**Results:**

Fifty-three unique miRNAs were identified (adjusted *p *< 0.05, fold of change >2), among which 32 miRNAs were upregulated and 21 miRNAs were downregulated in coronary artery disease patients. Reverse transcription quantitative PCR validated the results for seven miRNAs including miR-141-3p, miR-183-5p, miR-200a-5p, miR-205-5p, miR-429, miR-382-5p and miR-485-3p, with the last two downregulated. GO and KEGG analysis by mirPath indicated that these differentially expressed miRNAs were enriched in cell survival, apoptosis, proliferation, and differentiation.

**Conclusions:**

Coronary artery disease patients showed differential epicardial adipose tissue exosomal miRNA expression compared with patients without coronary artery disease. The results provide clues for further studies of mechanisms of atherosclerosis.

## 1. Introduction

Among noncommunicable diseases, cardiovascular disease is the leading cause of death worldwide; coronary artery disease contributes to most cardiovascular deaths [1, 2]. Coronary atherosclerosis, the most prominent feature of coronary artery disease, leads to lumen stenosis of coronary arteries and then myocardial ischemia [3]. Despite a great number of studies, the mechanisms of atherosclerosis are still unclear.

Epicardial adipose tissue (EAT) has emerged as a prevalent target of cardiovascular research. EAT is a visceral fat deposit between myocardium and visceral pericardium, which is mainly distributed in atrioventricular and interventricular grooves that surround the coronary arteries [4]. Although the weight of EAT varies, it can account for 20% of total heart weight in an average person [5]. More interestingly, there are no anatomical barriers between EAT and coronary arteries or myocardium because there is no fascia in between; thus, direct interaction is possible between EAT and coronary arteries or myocardium [4]. EAT is also a major source of proinflammatory cytokines such as interleukin-6, interleukin-10, and monocyte chemoattractant protein-1 as well as adipocytokines such as omentin, adiponectin, leptin, and vaspin. Thus, EAT has a significant activity in heart physiology and pathophysiology including coronary atherosclerosis [6]. In addition, EAT volume is independently associated with coronary events or major adverse cardiovascular events [7, 8]. However, still unclear are the exact mechanisms of EAT’s effects in coronary atherosclerosis.

Exosomes also have essential functions in the process of atherosclerosis. Exosomes are 30–150 nm lipid bilayer vesicles secreted by cells; exosomes contain bioactive substances such as nucleic acids, lipids, and proteins [9, 10]. Exosomes appear to function in cell-to-cell communication because they can transfer their contents between cells of different origins to participate in cellular signaling pathways [10]. Many studies provide evidence that exosomes are involved in atherosclerosis [11]. Exosomes transfer non-coding RNAs, cytokines, neutral lipids to endothelial cells, vascular smooth muscle cells, and macrophages involved in atherogenesis. Transfer of exosome materials induces apoptosis or activation or phenotypic transformation of cells, which results in atherosclerotic lesion initiation and progression [9]. It is unclear how exosomes affect cellular signal transduction during atherogenesis.

MicroRNAs (miRNAs) are crucial regulatory molecules in the pathogenesis of atherosclerosis [12]. MicroRNAs are small, single-stranded, non-coding RNAs that regulate protein synthesis by base pairing with and destabilizing their target mRNAs [12]. Parahuleva *et al*. [13] reported that atherosclerotic lesions and healthy arteries showed different miRNA expression profiles. Similarly, Fichtlscherer *et al*. [14] found different profiles of circulating miRNAs in patients with and without coronary artery disease. Lu *et al*. [15] reported that miRNAs influenced different cell types in contributing to atherosclerosis. Of note, according to Thomou *et al*. [16], adipose tissue was a main source of circulating exosomal miRNAs. Because exosomes differ between different adipose depot origins [16], and EAT shows a special transcriptomic signature [17], we anticipate that EAT has a specific exosomal miRNA profile.

The aim of this study was to profile exosomal miRNAs from EAT in patients with and without coronary artery disease and to identify candidate miRNAs for further studies on atherosclerosis. Prediction of miRNA target genes was performed by bioinformatic analysis to provide clues to signaling pathways involved in coronary atherosclerosis.

## 2. Materials and Methods 

### 2.1 Patients

We enrolled 24 patients who had undergone elective cardiac surgery. Before the surgery, coronary angiography was performed to confirm the status of coronary artery disease (CAD). According to the angiography results, the patients were put into a CAD group (n = 12) and a non-CAD (NCAD) group (n = 12). The CAD group was defined as patients undergoing off-pump coronary artery bypass grafting for three vessel disease, two-vessel disease with lesions at proximal left anterior descending artery or left main disease. The NCAD group was composed of patients who underwent open-heart surgery for mitral or aortic valve replacement or aortic arch replacement and angiography did not show significant coronary stenosis (no stenosis more than 50%). The key exclusion criteria were the following: age >80 years, acute myocardial infarction, autoimmune diseases, renal or liver failure, pharmacological glucocorticoid or immunosuppressive therapy, and history of percutaneous coronary intervention or open-heart surgery. According to Reviewer suggestions, we then enrolled eight patients in the CAD group and eight patients in NCAD Group to acquire exosomes to validation by reverse transcription quantitative PCR (RT-qPCR).

This study complied with the Declaration of Helsinki and was approved by the Ethics Committee of Beijing Anzhen Hospital, Capital Medical University. All patients provided signed informed consent.

### 2.2 Clinical Data Collection and Blood Sample Test 

Clinical data including demographic data, body weight, height, medical history, and examination were recorded on admission to the hospital and were obtained from the records of Beijing Anzhen Hospital, Capital Medical University (Beijing, China). BMI was calculated as weight (kg) divided by the square of height ($m^{2}$). Venous blood samples were obtained in sodium heparin Vacutainers (Becton–Dickinson) in a 12-hour fasting condition in the morning after admission; blood samples were transmitted to the central laboratory of Beijing Anzhen Hospital for measurements of lipid profiles, fasting glucose, creatinine, and blood urea nitrogen (BUN).

### 2.3 Epicardial Adipose Tissue Acquisition and Culture 

EAT biopsies (average 0.4 g) were harvested near the proximal right coronary artery before the initiation of the cardiopulmonary bypass and were transported to the laboratory as soon as possible. The adipose tissue biopsies were washed twice in sterilized phosphate-buffered saline (PBS) and then cut into small pieces (no more than 4 mm × 4 mm), followed by incubation for 24 hours in 100-mm petri dishes with 20 mL DMEM-F12 supplemented with 50 mg/mL penicillin and 50 IU/mL streptomycin without serum supplement.

### 2.4 Exosome Isolation 

Culture supernatants were collected and centrifuged at 800 g for 5 minutes and then centrifuged at 3000 g for 15 minutes. The supernatants were filtered through 0.22-μm membrane filters to remove cells and cell debris. The purified supernatants were then used to acquire exosome pellets. Exosomes were isolated by the Exosome Isolation Kit for Cell Culture Medium (polyethylene glycol precipitation method, Cat. No: E2120, WeiHui Biotech, Beijing, China) following manufacturer’s instructions.

### 2.5 Nanoparticle Tracking Analysis and Transmission Electron Microscopy 

The ZetaView System (Particle Matrix, Meersbusch, Germany), which uses a set of mirrors and lenses to focus a laser on the sample chamber, was used to determine exosome size distribution. The hydrodynamic radius of a single particle was determined by tracking its Brownian motion. An exosomal aliquot (20 μL) was loaded on to a formvar-carbon coated grid for 10 min, then negatively stained with 2% aqueous phosphotungstic acid and washed twice with 20 μL PBS on parafilm. Then the grid was scanned in a transmission electron microscope (JEM-1400plus, Japan).

### 2.6 Immunoblotting

Exosomal lysates from the CAD and NCAD groups and cultured EAT were prepared by treatment with Radio-Immunoprecipitation Assay (RIPA) buffer supplemented with a protease inhibitor mixture. Protein concentration was determined with the BCA Protein Assay Kit (Thermo Scientific, Waltham, MA, USA) Protein (20 μg) of each group was electrophoretically separated on SDS-polyacrylamide gels and transferred to polyvinylidene difluoride membranes. After blocking, the membranes were incubated with antibodies for exosomal marker CD81 (1:1000, ab109201, Abcam, UK), Alix (1:1000, #2171, CST, USA), Flotillin 1 (1:1000, ab41927, Abcam), CD 63 (1:1000, ab134045, Abcam, UK) and, as a negative control, Calnexin (1:1000, ab13504, Abcam, UK) and GAPDH (1:250,000, KM9002, Sanjian, China). After being washed with PBST, the blots were then incubated with horseradish peroxidase conjugated anti-rabbit IgG (1:2000, ZB-2301, ZSGB-BIO, Beijing, China) or anti-mouse IgG (1:2000, ZB-2305, ZSGB-BIO, Beijing, China) for 60 min at 37 ℃. Proteins were detected with the Pierce ECL Western Blotting Substrate (Thermo Scientific, USA).

### 2.7 Exosome RNA Isolation 

According to manufacturer’s recommendations, total exosomal RNA was isolated by the Exosomal RNA isolation kit (Cat. No: E1520-R, WeiHui Biotech, Beijing, China), which is based on the TRIzol method for RNA isolation and consists of N1, N2, N3 and N4 solutions, RNA precipitator and RNA elution buffer. Briefly, 250 μL N1 was mixed with 75–150 μL exosome sample thoroughly to denature the exosomes. Then, 50 μL N2 was added, followed by vortexing and centrifugation at 12,000 g for 15 min, and the upper phase was transferred to a fresh tube. Then, 125 μL N3 and 1 μL RNA precipitator were added, mixed thoroughly and centrifuged at 12,000 g for 10 min to obtain the RNA lysates. The RNA lysates were then washed with 250 μL N4 and centrifuged at 7500 g for 5 min to obtain the lysates twice, followed by elution in 15 μL RNA elution buffer. RNA quantification and quality were assessed by the Agilent Bioanalyzer 2100 system (Agilent Technologies, USA).

### 2.8 RNA Library Preparation and Sequencing

For each sample, 20 ng of total exosomal RNA was prepared as input material to construct a cDNA library of small RNAs. We used NEBNext Multiplex Small RNA Library Prep Set for Illumina (NEB, USA) to generate the sequencing libraries following manufacturer’s recommendations. Index codes were added to the RNAs. First strand cDNA synthesis was performed after ligation of 3’ and 5’ adaptors. The cDNA was amplified by PCR, and the PCR products were purified on 8% polyacrylamide gel (100V, 80 min) to obtain DNA fragments of 140–160 bp. Then the quality of the library was assessed on the Agilent Bioanalyzer 2100 system. Finally, the library preparations were sequenced on the Illumina Novaseq (6000) SE50 platform (Illumina, USA).

### 2.9 Differential Expression of miRNA

Transcripts per million (TPM) values were calculated to estimate miRNA expression. We used an R package to calculate differential expression for transcript level [18], with the *p*-values adjusted by the Benjamini & Hochberg method; adjusted *p* value < 0.05 and fold change >2.0 were set as the threshold for significantly different expression. Samples were arranged in groups by hierarchical clustering analysis based on TPM values, and a volcano plot was constructed.

### 2.10 Reverse Transcription Quantitative Polymerase Chain Reaction (RT-qPCR) Validation

To confirm the results obtained by high-throughput sequencing, we performed RT-qPCR validation of the top 10 significantly upregulated miRNAs and the top 10 downregulated miRNAs. After adding 25 fmol cel-miR39 to each exosome sample, total RNA was extracted from the exosomes and converted to cDNA (see earlier) with primers shown in **Supplementary Table 1**. RT-qPCR was performed on an ABI 7500 Fast System (Thermo, USA). Raw quantification of each sample was normalized to cel-39 with data calculated by the $2^{-\Delta \,\Delta \,\text{Ct}}$ method.

### 2.11 Gene Ontology and Pathway Analysis

In the stage of functional analysis, we selected qPCR-confirmed miRNAs to perform further analysis. MicroRNA target gene prediction was performed with the TarBase 7.0 database [19], a database of experimentally validated miRNA targets. Then, Gene Ontology (GO) enrichment and KEGG pathway enrichment of the predicted target genes were performed to investigate the functions and pathways of the target genes with miRPath V.3 platform [20].

### 2.12 Statistical Analysis 

Statistical analysis of the clinical baseline was accessed with SPSS 24.0 for Windows (IBM, NY, USA). Continuous data were represented as the mean ± SD or the median (lower quartile, upper quartile), as appropriate. Student’s *t*-test was performed to compare the mean values, and the Kruskal-Wallis H test was used to compare the median values. Categorical variables were represented as percent and analyzed by a Chi square test. In GO and KEGG analysis with mirPath, the false discovery rate was calculated by the Benjamini-Hochberg method. *p* or adjusted *p* or false discovery rate value <0.05 indicated statistical significance. Detailed statistical methods for each analysis are described above.

## 3. Results

### 3.1 Clinical Characteristics of Participants

Twenty-four patients who underwent elective open-heart surgery were enrolled in this study for RNA high-throughput sequencing (HTS group), and 16 patients were enrolled for RT-qPCR validation (qPCR group). Two patients in the RNA sequencing group were eventually omitted because of insufficient exosomal RNA. Tables 1,2 show the participant baseline characteristics. CAD patients were more likely to have been taking aspirin, β-blockers, and statins. There was no statistical difference in age, sex, body mass index, and blood pressure and no difference in hypertension and diabetes mellitus between the CAD group and non-CAD (NCAD) group. Blood sample test results also did not reveal any differences in leukocytes, hemoglobin, fasting blood glucose, total cholesterol, low-density lipoprotein cholesterol, high-density lipoprotein cholesterol, triglycerides, uric acid, blood urea nitrogen, and serum creatine. Preoperative echocardiography did not show any statistical difference in left ventricular end-diastolic dimension and left ventricular ejection fraction. These clinical characteristics appeared to be similar between HTS and qPCR groups.

**Table 1.** **Characteristics of the Patients in HTS Group**.

Characters	CAD (n = 11)	NCAD (n =11)	*p* Value
Age, years	64 ± 4	62 ± 7	0.267
Male, n (%)	6 (54.5)	6 (54.5)	1.000
BMI, kg/m²	24.4 ± 3.3	25.3 ± 3.1	0.519
Systolic blood pressure, mmHg	130 ± 8	124 ± 10	0.117
Diastolic blood pressure, mmHg	79 ± 7	74 ± 12	0.302
Hypertension, n, %	3 (27.3)	3 (27.3)	0.635
Diabetes, n, %	3 (27.3)	2 (18.2)	1.000
Leukocytes, ×${\mathrm{10}}^{9}$	6.3 ± 1.4	6.3 ± 2.1	0.980
Hemoglobin, g/L	134 ± 13	136 ± 13	0.759
Fasting blood glucose, mmol/L	5.39 (5.02, 8.57)	5.45 (5.38, 5.72)	0.922
Total cholesterol, mmol/L	3.9 ± 0.9	4.0 ± 1.0	0.825
LDL-C, mmol/L	2.2 ± 0.8	2.4 ± 0.9	0.523
HDL-C, mmol/L	0.98 (0.91, 1.10)	1.03 (0.94, 1.97)	0.324
Triglycerides, mmol/L	1.5 ± 0.5	1.1 ± 0.5	0.072
Uric acid	306.9 (259.4, 406.2)	359.6 (280.8 389.4)	0.974
BUN, mmol/L	5.28 (5.00, 6.38)	4.87 (3.92, 6.38)	0.393
Serum creatinine, μmmol/L	73.2 (57.3, 83.2)	72.0 (60.5, 88.4)	0.768
LVEDD, mm	47 ± 3	50 ± 9	0.312
LVEF, %	64 ± 6	64 ± 7	0.364
Aspirin, n, %	7 (63.6)	0 (0.0)	0.004
Clopidogrel, n, %	4 (36.3)	0 (0.0)	0.090
β-blocker, n, %	10 (90.9)	3 (27.3)	0.008
Statin, n, %	9 (81.8)	0 (0.0)	0.000
Oral antidiabetic drugs, n, %	2 (18.2)	2 (18.2)	1.000
CCB, n, %	2 (18.2)	2 (18.2)	1.000
Diuretics, n, %	3 (27.3)	6 (54.5)	0.387
ACEI/ARB, n, %	1 (9.1)	2 (18.2)	1.000

Data are presented as mean ± SD, median (low quartile, upper quartile), or percent. BMI, body mass index; LDL-C, low-density lipoprotein cholesterol; HDL-C, high-density lipoprotein cholesterol; BUN, blood urea nitrogen; LVEDD, left ventricular end-diastolic dimension; LVEF, left ventricular ejection fraction; CCB, calcium channel blockers; ACEI, angiotensin converting enzyme inhibitors; ARB, angiotensin receptor blockers; HTS, high throughput sequencing.

**Table 2.** **Characteristics of the Patients in qPCR Group**.

Characters	CAD (n = 8)	NCAD (n = 8)	*p *Value	*p* Value (HTS group vs. qPCR group)
Age, years	61 ± 11	56 ± 11	0.377	0.181
Male, n (%)	6 (75.0)	5 (62.5)	1.000	0.376
BMI, kg/m²	26.2 ± 1.4	24.4 ± 3.6	0.216	0.715
Systolic blood pressure, mmHg	133 ± 8	127 ± 23	0.511	0.482
Diastolic blood pressure, mmHg	81 ± 11	76 ± 16	0.421	0.712
Hypertension, n, %	6 (75.0)	4 (50.0)	0.608	0.030
Diabetes, n, %	3 (37.5)	1 (12.5)	0.569	1.000
Leukocytes, ×${\mathrm{10}}^{9}$	7.7 (5.0, 7.9)	5.8 (4.8, 7.2)	0.294	0.847
Hemoglobin, g/L	142 ± 13	143 ± 11	0.904	0.063
Fasting blood glucose, mmol/L	5.39 (4.67, 8.22)	4.78 (4.52, 5.34)	0.189	0.193
Total cholesterol, mmol/L	3.9 ± 0.9	4.2 ± 0.5	0.454	0.786
LDL-C, mmol/L	2.2 ± 0.7	2.3 ± 0.3	0.660	0.860
HDL-C, mmol/L	0.98 (0.83, 1.14)	1.27 (0.94, 1.34)	0.093	0.988
Triglycerides, mmol/L	1.7 ± 0.7	1.1 ± 0.3	0.041	0.856
Uric acid	357.2 ± 38.8	330.5 ± 71.0	0.371	0.788
BUN, mmol/L	6.34 ± 2.17	4.85 ± 1.24	0.112	0.574
Serum creatinine, μmmol/L	86.3 ± 18.6	76.2 ± 10.8	0.612	0.522
LVEDD, mm	47.5 (44.0, 48.8)	47.0 (44.5, 49.5)	0.873	0.888
LVEF, %	62.5 (58.5, 65.3)	65.0 (58.5, 65.3)	0.490	0.349
Aspirin, n, %	7 (87.5)	0 (0.0)	0.001	0.567
Clopidogrel, n, %	6 (75.0)	0 (0.0)	0.007	0.267
β-blocker, n, %	7 (87.5)	2 (25.0)	0.041	0.861
Statin, n, %	7 (87.5)	1 (12.5)	0.010	0.578
Oral antidiabetic drugs, n, %	2 (25.0)	0 (0.0)	0.467	1.000
CCB, n, %	2 (25.0)	0 (0.0)	0.467	1.000
Diuretics, n, %	1 (12.5)	3 (37.5)	0.569	0.307
ACEI/ARB, n, %	2 (25.0)	0 (0.0)	0.467	1.000

Data are presented as mean ± SD, median (low quartile, upper quartile), or percent. BMI, body mass index; LDL-C, low-density lipoprotein cholesterol; HDL-C, high-density lipoprotein cholesterol; BUN, blood urea nitrogen; LVEDD, left ventricular end-diastolic dimension; LVEF, left ventricular ejection fraction; CCB, calcium channel blockers; ACEI, angiotensin converting enzyme inhibitors; ARB, angiotensin receptor blockers; HTS, high throughput sequencing.

### 3.2 Characterization of Exosomes Derived from EAT in CAD and NCAD Patients 

**Fig. 1.**
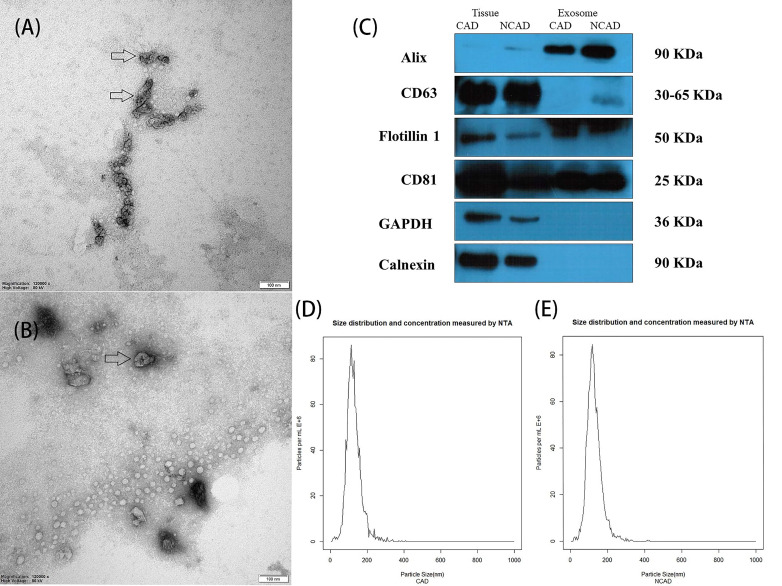
**Identification of exosomes by transmission electron microscopy (TEM), immunoblotting, and nanoparticle tracking analysis**. TEM showed that exosomes derived from EAT in CAD patients (A) and NCAD patients (B) had a saucer-like shape with a lipid bilayer. Arrowheads point to exosomes. Scale bar = 100 nm. (C) Immunoblot showed that exosomes from CAD and NCAD patients were positive for exosomal marker Alix, CD 81, and Flotillin 1, and negative for non-exosome markers Calnexin and GAPDH. Tissue (EAT) was used as a control for the negative markers. Concentration and size of exosomes in CAD (D) and NCAD (E) groups were analyzed by nanoparticle tracking analysis.

Exosomes derived from EAT were isolated by their unique size and density. Fig. 1A,B show that the exosomes derived from EAT in CAD patients and NCAD patients had a cup-like shape and were similar in size (20–150 nm). The presence of exosomal identity markers CD81, Flotillin 1, and Alix, and the absence of negative exosomal marker Calnexin, was confirmed by immunoblotting (Fig. 1C). Nanoparticle tracking analysis showed that the size of most small vesicles was 60–150 nm (Fig. 1D,E). All these verification tests confirmed that the isolated small vesicles were exosomes.

### 3.3 Exosomal miRNAs Identification 

The raw data is available on Sequence Read Archive (SRA) platform of NCBI with access ID PRJNA698758. We found 1489 miRNAs by sequencing after low-quality reads and removal of contaminants and adaptor sequences. The clean miRNA reads of high-throughput sequencing were then compared with miRbase 20.0 to look for known miRNAs. In **Supplementary Fig. 1**, we list the top 30 most abundant miRNAs in EAT-derived exosomes by shinyCircos [21].

**Fig. 2.**
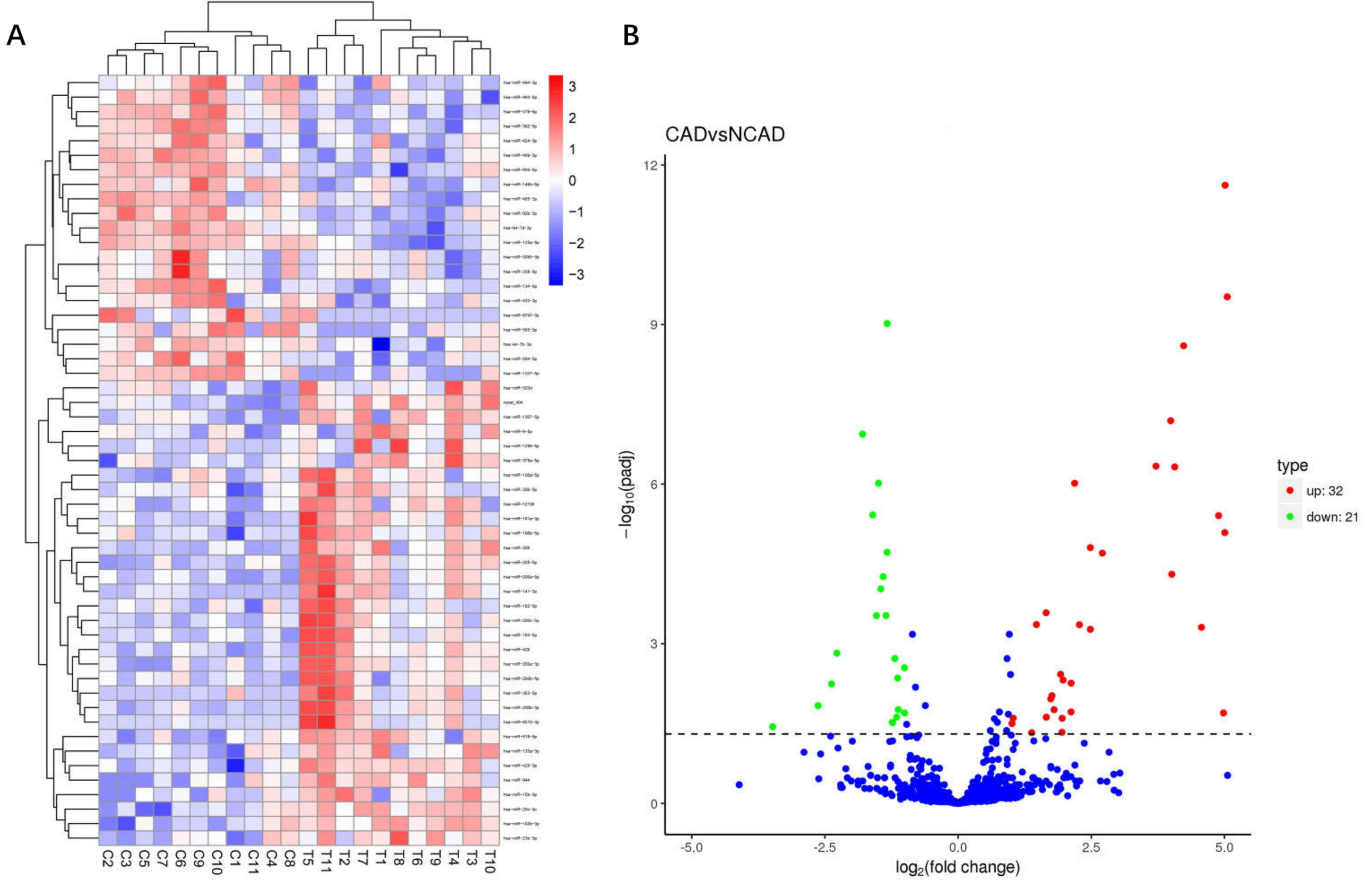
**Differentially expressed miRNAs in exosomes derived from EAT from CAD and NCAD individuals**. (A) Hierarchical clustering for differentially expressed miRNAs in CAD (n = 11) versus NCAD (n = 11) (adjusted *p *< 0.05 and fold change >2). Columns show the clustering of exosome samples, in which C1-C11 refer to NCAD patients, and T1-T11 refer to CAD patients. Rows display the clustering of genes. Red or blue represents upregulated or downregulated miRNAs, respectively. (B) Volcano plot of the substantially differentially expressed miRNAs shows 32 miRNAs were upregulated and 21 miRNAs were downregulated.

We identified exosomal miRNAs that were differentially expressed between CAD and NCAD samples (Fig. 2). There were 53 unique miRNAs (adjusted *p <* 0.05, fold of change >2), among which 32 miRNAs were upregulated, including an unknown miRNA, and 21 miRNAs were downregulated in CAD patients. The miR-200 families and miR-206 were the most upregulated miRNAs, and miR-379-5p, let-7d-3p, miR-146b-5p, and miR-92b-3p were the most downregulated miRNAs.

### 3.4 RT-qPCR Validation

**Fig. 3.**
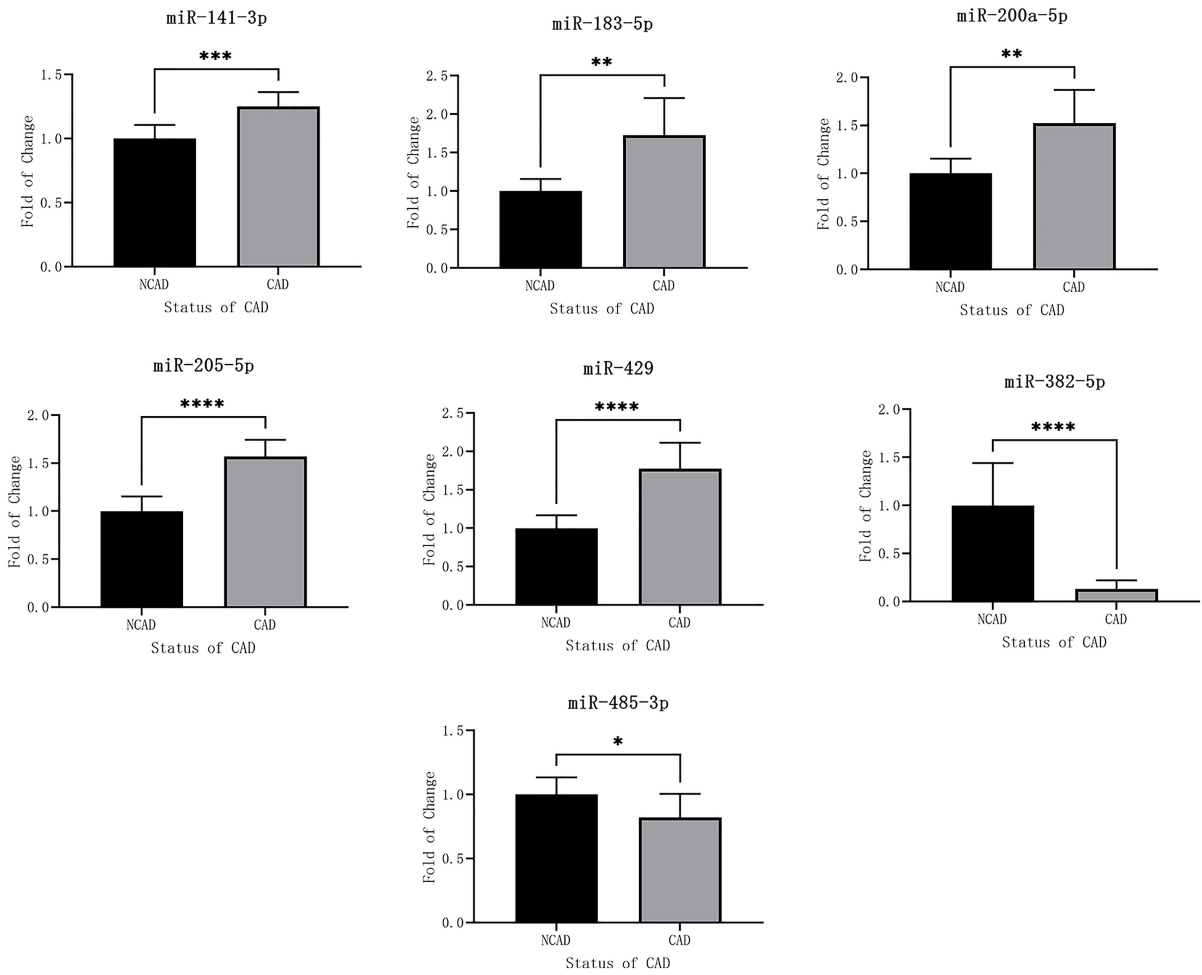
**RT-qPCR results of seven miRNAs**. * 0.01 < *p *< 0.05, ** 0.001 <* p *< 0.01, *** 0.0001 <* p *< 0.001, **** *p *< 0.0001.

Fig. 3 shows the expression levels of seven confirmed miRNAs of 20 candidates. Five miRNAs were upregulated and two were downregulated in patients with CAD, a result that was consistent with sequencing results.

### 3.5 Functional Analysis 

**Fig. 4.**
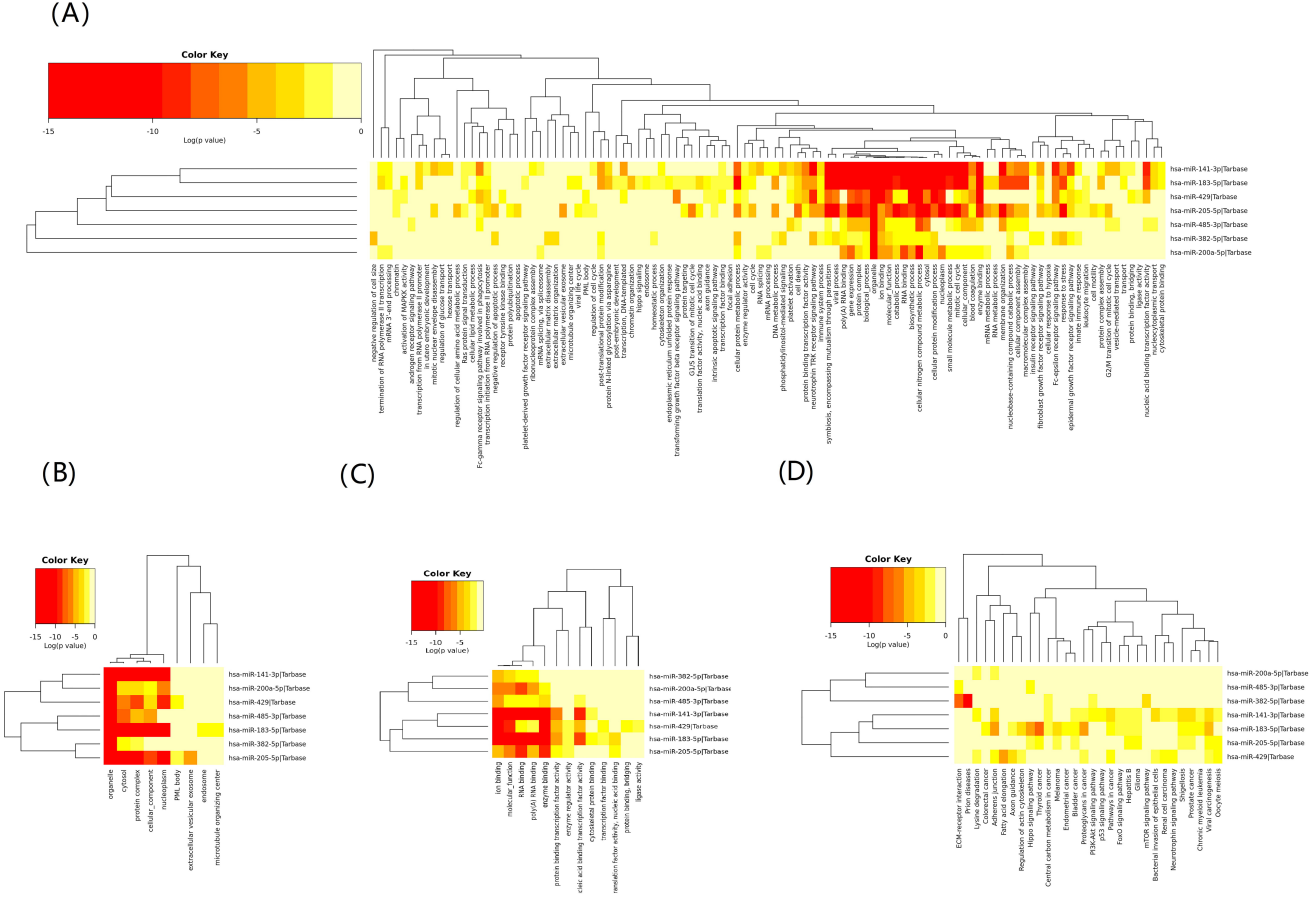
**GO (A–C) and KEGG (D) analysis**. (A) Biological processes (BP), (B) molecular functions (MF), (C) cellular components (CC). The x-axis displays enriched GO biological process terms or KEGG pathways, and the y-axis refers to miRNA targets. The color of each square represents the significance of enrichment. GO, Gene Ontology; KEGG, Kyoto Encyclopedia of Genes and Genomes.

To assess possible regulatory mechanisms of exosomal miRNA, we used Tarbase 7.0 to select target genes of the confirmed miRNAs that were differentially expressed between the CAD and NCAD patients. The results were used for functional analysis with the GO and KEGG databases and pathway analysis by miRPath. The detailed results are presented in Figure. By GO analysis (Fig. 4A–C), we investigated biological processes (BP), molecular functions (MF) and cellular components (CC). KEGG analysis (Fig. 4D) indicated that significantly enriched pathways were axon guidance, pathways in cancer, PI3K-Akt signaling, and the FoxO signaling pathway (FDR <0.05). Fig. 4D also showed that miR-382-5p and miR-141-3p were involved in the PI3K-Akt signaling pathway and that miR-183-5p and miR-141-3p were involved in the FoxO signaling pathway. GO analysis indicated that miR-183-5p was also relevant to focal adhesion (Fig. 4A). These results indicated that these differentially expressed miRNAs were enriched in cell survival, apoptosis, proliferation, and differentiation.

## 4. Discussion

Because of its unique anatomical features and active biological characteristics, EAT shows a close relationship with coronary atherosclerosis. Exosomes have been considered relevant in coronary artery disease, and exosomes derived from visceral adipose tissue showed a proatherogenic effect [22]. Our study appears to be the first to describe microRNA expression profiles of EAT-derived exosomes in CAD. We identified 53 differently expressed miRNAs, 21 that were downregulated, and the remainder upregulated. Seven miRNAs were confirmed in qPCR validation.

Many studies confirmed that exosomes from EAT could target vessel cells. Xie and colleagues [22] and our team [23] both confirmed intake of adipose-derived exosomes in macrophages. Briefly, Xie *et al*. [22] found that visceral adipose-derived exosomes from high-fat diet-induced obese mice promoted macrophage polarization and foam cell formation. We showed previously that perivascular adipose tissue-derived exosomes could reduce macrophage foam cell formation by regulating expression of cholesterol transporters. Shaihov-Teper *et al*. [24] showed that extracellular vesicles from EAT targeted endothelial cells and facilitated angiogenesis.

In this study, miR-200 family members were the most differentially expressed miRNAs among the upregulated miRNAs, especially miR-141-3p, miR-200a-5p and miR-429 validated by qPCR. The miR-200 family, miR-200a, miR-200b, miR200c, miR-141, and miR-429, exerts a pro-inflammatory function in the process of atherosclerosis [25]. The miR-200 family can be upregulated during oxidative stress of endothelial cells [26], and Zhang *et al*. [27] reported that increased miR-429 may target Bcl-2 and induce endothelial cell apoptosis, which can be associated with atherosclerosis. In mice with type 2 diabetes, miR-429, miR-200b and miR-200c expression levels were elevated in vascular smooth muscle cells (VSMCs), and miR-200 mimics promoted monocyte-binding of VSMCs, which was reversed by miR-200 inhibitors [25]. In addition, Gong *et al*. [28] reported that miR-141-3p/miR-200a-3p might accelerate atherosclerosis by targeting Coiled-Coil Domain Containing 80 (CCDC80) in VSMCs. Another validated upregulated exosomal miRNA in our study was miR-205-5p. Meng *et al*. [29] found that miR-205-5p promoted unstable atherogenesis and suppressed cholesterol efflux, which could result in susceptibility to free cholesterol-triggered macrophage apoptosis. Son *et al*. [30] reported that murine-specific miR-712 could promote endothelial inflammation and accelerate the process of atherosclerosis; interestingly, miR-205 shares the most sequence with miR-712 and might be a homologue of miR-712.

In our study, miR-485-3p and miR-382-5p were confirmed to be less prevalent in EAT-derived exosomes from CAD patients. MiR-485-5p was found to decrease the expression of target gene MMP14 to inhibit epithelial-mesenchymal transition (EMT) [31], and, interestingly, EMT commonly occurs in atherosclerotic plaques and may induce plaque instability [32]. Hu *et al*. [33] reported that overexpression of miR-382-5p inhibited nuclear factor IA expression and proinflammatory cytokines levels including IL-6, IL-1β and TNF-α, which might have a beneficial function in atherosclerosis deterioration.

In GO analysis, focal adhesion was one of the significant terms among predicted target genes of selected miRNAs. This finding suggested that focal adhesion has a vital function in atherosclerosis. Focal adhesion is the adhering junction between cell and extracellular matrix (ECM), providing communication between cytoskeleton and ECM and making possible interactions between vascular wall and extracellular environment [34]. Tsai *et al*. [35] showed that deficiency of Galectin-1 attenuated FA formation by VSMCs, disclosing that Galectin-1 promotes focal adhesion turnover which results in restriction of the motility of VSMCs that suppresses neointimal formation after vascular injuries. Focal adhesion kinase (FAK) is a key constituent of focal adhesion [34], and inhibition of FAK catalytic activity induces its nuclear enrichment which blocks neointimal hyperplasia and VMSC proliferation by modulating the GATA-binding protein 4-cyclin D1 signal pathway in vascular injury [36].

In KEGG analysis, the PI3K/Akt signaling pathway was a significant KEGG pathway of predicted target genes of selected miRNAs. The PI3K/Akt pathway is common in atherogenesis because many signals are transduced by this pathway in atherosclerosis. The PI3K/Akt pathway has an important function in macrophage proliferation, survival migration and polarization that might impact atherosclerosis [37]. The PI3K/Akt pathway was also detected in endothelial cell inflammation and injury [38] and could induce VSMC foam cell formation and lipid accumulation [39], which indicated the widespread effects of the PI3K/Akt signaling pathway on pathology of atherosclerosis.

FoxO (the O subfamily of forkhead) signaling pathway was another enriched KEGG pathway of predicted target genes. The FoxO family, FoxO1, 3, 4, and 6, is involved in homeostasis in endothelial cells [40]. FoxO1 is coupled with metabolic status and survival of cells in the cardiovascular system [41], and its regulation is of vital importance because an imbalance can be detrimental [41]. Wilhelm *et al*. [42] reported that FoxO1 inhibited Myc, resulting in quiescence of endothelial cells, which might support endothelial survival. However, in vascular endothelial cells of FoxO knock-out mice, inflammatory cytokines IL-1β, IL-6, and monocyte chemotactic protein 1, and reactive oxygen species were reduced, whereas nitric oxide generation was elevated, which indicated that FoxO inhibition might pose an atheroprotective effect [43]. Moreover, Deng *et al*. [44] reported that FoxO1/3 inhibition boosted VSMC calcification by the phosphatase and tensin homolog/AKT pathway. More interestingly, in visceral adipose tissue of endothelial cell-FoxO1 knock-out mice, vascular density increased, and, when fed a high-fat diet, these mice exhibited more microvascular remodeling in visceral adipose tissue and showed less adipose accumulation with adaptive metabolic change of endothelial cells [45]. We wonder whether the same effect can occur on arteries by visceral adipose tissue including EAT by FoxO signaling just like microvascular circulation.

Exosomes can be a source of miRNAs; the differentially expressed miRNA in our study have already been found to express in extracellular vesicles (EVs). However, this could not indicate that these findings were useless. We know that the exosomes or EVs in serum and other body fluids are from all types of tissues and cells, with most exosomes stemming from adipose tissue. But different tissues can release different exosomes, and even adipose tissue from different positions can release different exosomes; thus, we cannot tell the origin of exosomes without specific research [16]. Exosomal communication is more likely to take occur by paracrine regulation, whereas circulatory exosomes do not have this potential. Because many miRNAs were found to take part in atherosclerosis in many cell types, such as macrophages, smooth muscle cells, and endothelial cells, it might be helpful to find the miRNAs’ origins because they might be targets for cures of atherosclerosis and markers for disease.

This study had some limitations. First, we could not select completely healthy controls because of ethical and practical issues; thus, we might not have ruled out all the confounding factors. Second, the enrolled patients were Chinese, and our findings may or may not extend to other ethnicities. Third, the sequencing results were too numerous to validate each one by RT-qPCR. Fourth, because the enriched terms and pathways were analyzed by bioinformatics, we could not provide direct detailed pathways in atherosclerosis; additional experimental testing should be performed.

## 5. Conclusions 

We obtained exosomes from EAT in CAD patients and NCAD controls and used high-throughput sequencing to acquire differential expression profiles for miRNAs. CAD patients showed different EAT exosomal miRNA expression profiles compared with NCAD patients. GO and KEGG analysis of predicted miRNA target genes was performed. The results provided clues for further studies of exosomal mechanisms of atherosclerosis.

## Data Availability

The datasets used and/or analyzed during this study are available from the corresponding author on reasonable request.

## Supplementary Material

Supplementary material associated with this article can be found, in the online version, at https://doi.org/10.31083/j.rcm2306206.

## References

[1] Benjamin EJ, Muntner P, Alonso A, Bittencourt MS, Callaway CW, Carson AP (2019). Heart Disease and Stroke Statistics-2019 Update: A Report from the American Heart Association. *Circulation*.

[2] Fuster V (2014). Global Burden of Cardiovascular Disease. *Journal of the American College of Cardiology*.

[3] Yahagi K, Kolodgie FD, Otsuka F, Finn AV, Davis HR, Joner M (2016). Pathophysiology of native coronary, vein graft, and in-stent atherosclerosis. *Nature Reviews Cardiology*.

[4] Iacobellis G (2015). Local and systemic effects of the multifaceted epicardial adipose tissue depot. *Nature Reviews Endocrinology*.

[5] Gaborit B, Sengenes C, Ancel P, Jacquier A, Dutour A (2017). Role of Epicardial Adipose Tissue in Health and Disease: A Matter of Fat. *Comprehensive Physiology*.

[6] Iacobellis G, Bianco AC (2011). Epicardial adipose tissue: emerging physiological, pathophysiological and clinical features. *Trends in Endocrinology & Metabolism*.

[7] Christensen RH, von Scholten BJ, Hansen CS, Jensen MT, Vilsbøll T, Rossing P (2019). Epicardial adipose tissue predicts incident cardiovascular disease and mortality in patients with type 2 diabetes. *Cardiovascular Diabetology*.

[8] Toya T, Corban MT, Imamura K, Bois JP, Gulati R, Oh JK (2020). Coronary perivascular epicardial adipose tissue and major adverse cardiovascular events after ST segment-elevation myocardial infarction. *Atherosclerosis*.

[9] Wang Y, Xie Y, Zhang A, Wang M, Fang Z, Zhang J (2019). Exosomes: An emerging factor in atherosclerosis. *Biomedicine & Pharmacotherapy*.

[10] Zhang J, Li S, Li L, Li M, Guo C, Yao J (2015). Exosome and Exosomal MicroRNA: Trafficking, Sorting, and Function. *Genomics, Proteomics & Bioinformatics*.

[11] Huber HJ, Holvoet P (2015). Exosomes: emerging roles in communication between blood cells and vascular tissues during atherosclerosis. *Current Opinion in Lipidology*.

[12] Feinberg MW, Moore KJ (2016). MicroRNA Regulation of Atherosclerosis. *Circulation Research*.

[13] Parahuleva MS, Lipps C, Parviz B, Hölschermann H, Schieffer B, Schulz R (2018). MicroRNA expression profile of human advanced coronary atherosclerotic plaques. *Scientific Reports*.

[14] Fichtlscherer S, De Rosa S, Fox H, Schwietz T, Fischer A, Liebetrau C (2010). Circulating MicroRNAs in Patients with Coronary Artery Disease. *Circulation Research*.

[15] Lu Y, Thavarajah T, Gu W, Cai J, Xu Q (2018). Impact of miRNA in Atherosclerosis. *Arteriosclerosis, Thrombosis, and Vascular Biology*.

[16] Thomou T, Mori MA, Dreyfuss JM, Konishi M, Sakaguchi M, Wolfrum C (2017). Adipose-derived circulating miRNAs regulate gene expression in other tissues. *Nature*.

[17] Schleinitz D, Krause K, Wohland T, Gebhardt C, Linder N, Stumvoll M (2020). Identification of distinct transcriptome signatures of human adipose tissue from fifteen depots. *European Journal of Human Genetics*.

[18] Love MI, Huber W, Anders S (2014). Moderated estimation of fold change and dispersion for RNA-seq data with DESeq2. *Genome Biology*.

[19] Vlachos IS, Paraskevopoulou MD, Karagkouni D, Georgakilas G, Vergoulis T, Kanellos I (2015). DIANA-TarBase v7.0: indexing more than half a million experimentally supported miRNA: mRNA interactions. *Nucleic Acids Research*.

[20] Vlachos IS, Zagganas K, Paraskevopoulou MD, Georgakilas G, Karagkouni D, Vergoulis T (2015). DIANA-miRPath v3.0: deciphering microRNA function with experimental support. *Nucleic Acids Research*.

[21] Yu Y, Ouyang Y, Yao W (2018). ShinyCircos: an R/Shiny application for interactive creation of Circos plot. *Bioinformatics*.

[22] Xie Z, Wang X, Liu X, Du H, Sun C, Shao X (2018). Adipose‐Derived Exosomes Exert Proatherogenic Effects by Regulating Macrophage Foam Cell Formation and Polarization. *Journal of the American Heart Association*.

[23] Liu Y, Sun Y, Lin X, Zhang D, Hu C, Liu J (2021). Perivascular Adipose-Derived Exosomes Reduce Foam Cell Formation by Regulating Expression of Cholesterol Transporters. *Frontiers in Cardiovascular Medicine*.

[24] Shaihov-Teper O, Ram E, Ballan N, Brzezinski RY, Naftali-Shani N, Masoud R (2021). Extracellular Vesicles from Epicardial Fat Facilitate Atrial Fibrillation. *Circulation*.

[25] Reddy MA, Jin W, Villeneuve L, Wang M, Lanting L, Todorov I (2012). Pro-Inflammatory Role of MicroRNA-200 in Vascular Smooth Muscle Cells from Diabetic Mice. *Arteriosclerosis, Thrombosis, and Vascular Biology*.

[26] Magenta A, Greco S, Gaetano C, Martelli F (2013). Oxidative Stress and MicroRNAs in Vascular Diseases. *International Journal of Molecular Sciences*.

[27] Zhang T, Tian F, Wang J, Jing J, Zhou S, Chen Y (2015). Atherosclerosis-Associated Endothelial Cell Apoptosis by MiR-429-Mediated down Regulation of Bcl-2. *Cellular Physiology and Biochemistry*.

[28] Gong D, Zhao ZW, Zhang Q, Yu XH, Wang G, Zou J (2020). The Long Noncoding RNA Metastasis-Associated Lung Adenocarcinoma Transcript-1 Regulates CCDC80 Expression by Targeting miR-141-3p/miR-200a-3p in Vascular Smooth Muscle Cells. *Journal of Cardiovascular Pharmacology*.

[29] Meng X, Yin J, Yu X, Guo Y (2020). MicroRNA-205-5p Promotes Unstable Atherosclerotic Plaque Formation in Vivo. *Cardiovascular Drugs and Therapy*.

[30] Son DJ, Kumar S, Takabe W, Woo Kim C, Ni C, Alberts-Grill N (2013). The atypical mechanosensitive microRNA-712 derived from pre-ribosomal RNA induces endothelial inflammation and atherosclerosis. *Nature Communications*.

[31] Liu H, Hu G, Wang Z, Liu Q, Zhang J, Chen Y (2020). CircPTCH1 promotes invasion and metastasis in renal cell carcinoma via regulating miR-485-5p/MMP14 axis. *Theranostics*.

[32] Evrard SM, Lecce L, Michelis KC, Nomura-Kitabayashi A, Pandey G, Purushothaman K (2016). Endothelial to mesenchymal transition is common in atherosclerotic lesions and is associated with plaque instability. *Nature Communications*.

[33] Hu Y, Zhao J, Li S, Huang J, Qiu Y, Ma X (2015). RP5-833a20.1/miR-382-5p/NFIA–Dependent Signal Transduction Pathway Contributes to the Regulation of Cholesterol Homeostasis and Inflammatory Reaction. *Arteriosclerosis, Thrombosis, and Vascular Biology*.

[34] Romer LH, Birukov KG, Garcia JGN (2006). Focal Adhesions. *Circulation Research*.

[35] Tsai M, Chiang M, Tsai D, Yang C, Hou H, Li Y (2018). Galectin-1 Restricts Vascular Smooth Muscle Cell Motility via Modulating Adhesion Force and Focal Adhesion Dynamics. *Scientific Reports*.

[36] Jeong K, Kim J, Murphy JM, Park H, Kim S, Rodriguez YAR (2019). Nuclear Focal Adhesion Kinase Controls Vascular Smooth Muscle Cell Proliferation and Neointimal Hyperplasia through GATA4-Mediated Cyclin D1 Transcription. *Circulation Research*.

[37] Linton MF, Moslehi JJ, Babaev VR (2019). Akt Signaling in Macrophage Polarization, Survival, and Atherosclerosis. *International Journal of Molecular Sciences*.

[38] Liu Y, Tie L (2019). Apolipoprotein M and sphingosine-1-phosphate complex alleviates TNF-α-induced endothelial cell injury and inflammation through PI3K/AKT signaling pathway. *BMC Cardiovascular Disorders*.

[39] Pi S, Mao L, Chen J, Shi H, Liu Y, Guo X (2021). The P2RY12 receptor promotes VSMC-derived foam cell formation by inhibiting autophagy in advanced atherosclerosis. *Autophagy*.

[40] Paik J, Kollipara R, Chu G, Ji H, Xiao Y, Ding Z (2007). FoxOs are Lineage-Restricted Redundant Tumor Suppressors and Regulate Endothelial Cell Homeostasis. *Cell*.

[41] Puthanveetil P, Wan A, Rodrigues B (2013). FoxO1 is crucial for sustaining cardiomyocyte metabolism and cell survival. *Cardiovascular Research*.

[42] Wilhelm K, Happel K, Eelen G, Schoors S, Oellerich MF, Lim R (2016). FOXO1 couples metabolic activity and growth state in the vascular endothelium. *Nature*.

[43] Tsuchiya K, Tanaka J, Shuiqing Y, Welch C, DePinho R, Tabas I (2012). FoxOs Integrate Pleiotropic Actions of Insulin in Vascular Endothelium to Protect Mice from Atherosclerosis. *Cell Metabolism*.

[44] Deng L, Huang L, Sun Y, Heath JM, Wu H, Chen Y (2015). Inhibition of FOXO1/3 Promotes Vascular Calcification. *Arteriosclerosis, Thrombosis, and Vascular Biology*.

[45] Rudnicki M, Abdifarkosh G, Nwadozi E, Ramos SV, Makki A, Sepa-Kishi DM (2018). Endothelial-specific FoxO1 depletion prevents obesity-related disorders by increasing vascular metabolism and growth. *eLife*.

