# Supplementary material for: MicroRNA Expression Profiles of Epicardial Adipose Tissue-Derived Exosomes in Patients with Coronary Atherosclerosis
Source: Rev Cardiovasc Med. 2022 May 31;23(6):206. doi: 10.31083/j.rcm2306206 (PMC11273655; doi:10.31083/j.rcm2306206)
Supplement: Supplementary file 1 [file 2153-8174-23-6-206-s1.zip › 2153-8174-23-6-206-s1.doc]

Supplementary Table 1. Primers of miRNAs in qPCR.

| Primer | Sequence (5' to 3') |
| --- | --- |
| hsa-miR-206 Forward | TGGAATGTAAGGAAGTGTGTGG |
| hsa-miR-429 Forward | TAATACTGTCTGGTAAAACCGT |
| hsa-miR-200b-3p Forward | TAATACTGCCTGGTAATGATGA |
| hsa-miR-200a-3p Forward | TAACACTGTCTGGTAACGATGT |
| hsa-miR-200a-5p Forward | CATCTTACCGGACAGTGCTGGA |
| hsa-miR-205-5p Forward | TCCTTCATTCCACCGGAGTCTG |
| hsa-miR-141-3p Forward | TAACACTGTCTGGTAAAGATGG |
| hsa-miR-181a-3p Forward | ACCATCGACCGTTGATTGTACC |
| hsa-miR-183-5p Forward | TATGGCACTGGTAGAATTCACT |
| hsa-miR-425-3p Forward | ATCGGGAATGTCGTGTCCGCCC |
| hsa-miR-379-5p Forward | TGGTAGACTATGGAACGTAGG |
| hsa-let-7d-3p Forward | CTATACGACCTGCTGCCTTTCT |
| hsa-miR-146b-5p Forward | TGAGAACTGAATTCCATAGGCTG |
| hsa-miR-92b-3p Forward | TATTGCACTCGTCCCGGCCTCC |
| hsa-miR-125a-5p Forward | TCCCTGAGACCCTTTAACCTGTGA |
| hsa-miR-382-5p Forward | GAAGTTGTTCGTGGTGGATTCG |
| hsa-miR-409-3p Forward | GAATGTTGCTCGGTGAACCCCT |
| hsa-miR-493-5p Forward | TTGTACATGGTAGGCTTTCATT |
| hsa-miR-654-5p Forward | TGGTGGGCCGCAGAACATGTGC |
| hsa-miR-485-3p Forward | GTCATACACGGCTCTCCTCTCT |
| cel-miR-39 Forward | ATGGTTCGTGGGTCACCGGGTGTAAATC |
| cel-miR-39 Reverse | GCAGGGTCCGAGGTATTC |

**Supplementary Fig. 1.** **Top 30 miRNAs in EAT-derived exosomes and microRNA genomic distribution.**
